# Supplementary material for: Saccharomyces boulardii (CNCM I-745) improves intestinal damage in sepsis by remodeling intestinal flora structure
Source: Front Cell Infect Microbiol. 2025 Jul 31;15:1602792. doi: 10.3389/fcimb.2025.1602792 (PMC12350329; doi:10.3389/fcimb.2025.1602792)
Supplement: Supplementary file 1 [file DataSheet1.zip › summary/summary/6_Taxonomy_community/1_Taxonomy_profiling/2_Phylum/Group/Phylum_abund_top30_Group.heatmap.pdf]

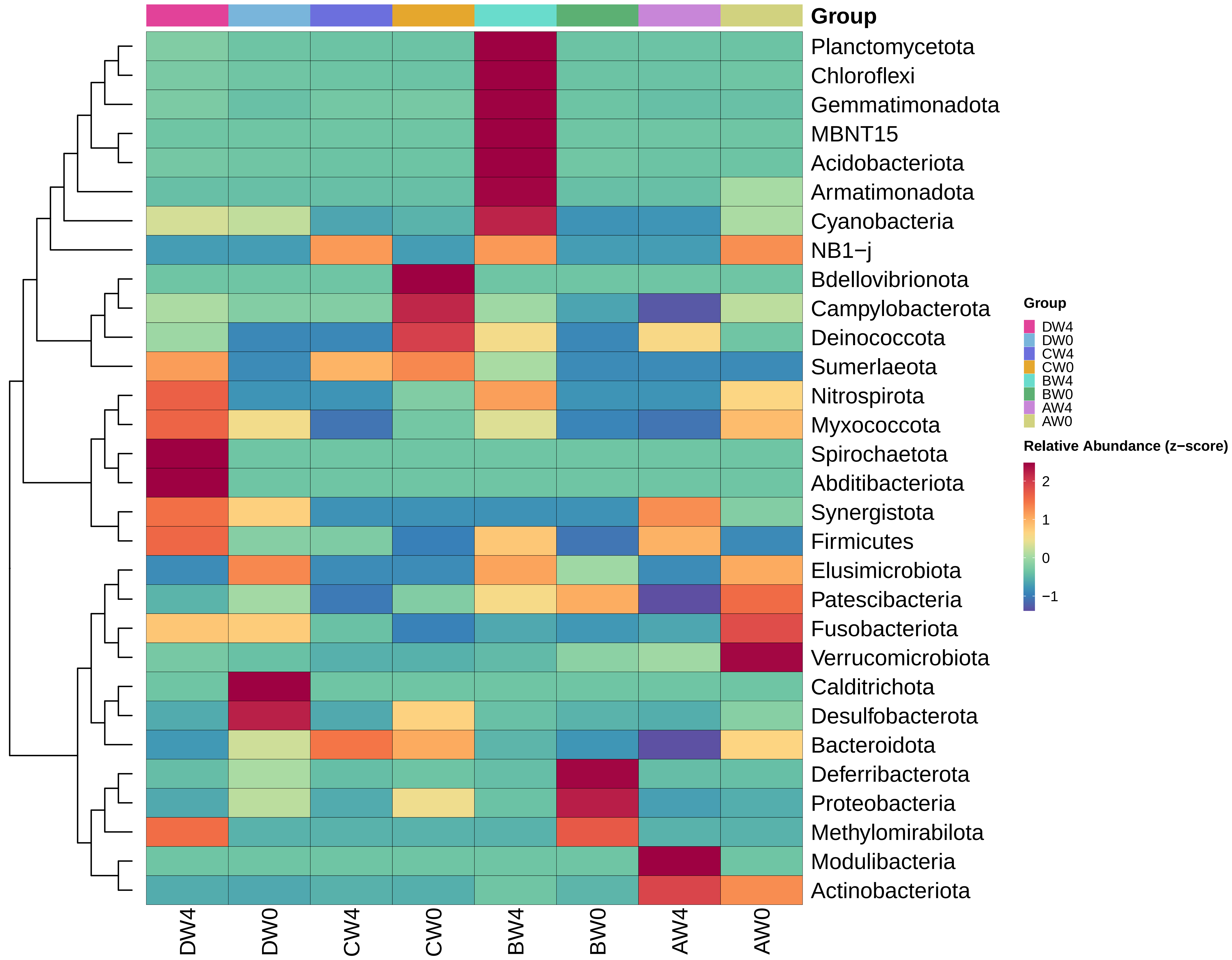
Group
Planctomycetota
Chloroflexi
Gemmatimonadota
MBNT15
Acidobacteriota
Armatimonadota
Cyanobacteria
NB1−j
Bdellovibrionota
Campylobacterota
Deinococcota
Sumerlaeota
Nitrospirota
Myxococcota
Spirochaetota
Abditibacteriota
Synergistota
Firmicutes
Elusimicrobiota
Patescibacteria
Fusobacteriota
Verrucomicrobiota
Calditrichota
Desulfobacterota
Bacteroidota
Deferribacterota
Proteobacteria
Methylomirabilota
Modulibacteria
Actinobacteriota
DW4
DW0
CW4
CW0
BW4
BW0
AW4
AW0
Group
DW4
DW0
CW4
CW0
BW4
BW0
AW4
AW0
Relative Abundance (z−score)
2
1
0
−1
